# Diagnosis and Treatment of Human Salmonellosis in Addis Ababa City, Ethiopia

**DOI:** 10.1155/2018/6406405

**Published:** 2018-05-23

**Authors:** Legesse Garedew, Semaria Solomon, Yoseph Worku, Hilina Worku, Debela Gemeda, Gada Lelissa, Yeshwondm Mamuye, Rajiha Abubeker, Amete Mihret, Surafel Fentaw, Abeibe Worku, Melish Bahiru, Girume Erenso

**Affiliations:** ^1^St Paul's Hospital Millennium Medical College, Addis Ababa, Ethiopia; ^2^Ethiopian Public Health Institute, Addis Ababa, Ethiopia; ^3^Alem Tena Clinic, Addis Ababa, Ethiopia; ^4^Selam Clinic, Addis Ababa, Ethiopia

## Abstract

**Background:**

Diagnosis using reliable tools and treatment following* in vitro *antimicrobial susceptibility tests are critical to proper addressing of antibiotic-resistant* Salmonella* infection.

**Methodology:**

A cross-sectional study was conducted to assess the practice of diagnosis and treatment of salmonellosis in Addis Ababa. Tube Widal test (for blood samples only), culture, biochemical and carbohydrate fermentation, serotyping, and antimicrobial susceptibility tests were employed for both blood and stool samples.

**Results:**

Of all the diseases listed in the diagnosis, nontyphoidal (*n* = 72, 13.71%) and typhoidal (*n* = 47, 8.95%) salmonellosis were the second and third common diseases. Among the 288 blood samples, almost half were positive for O, H, or both antigens. However, only 1 (0.68%) of the positive blood samples yielded* Salmonella* isolate during culture. The study demonstrated low specificity (0.68%) and positive predictive value (48.78%) of Widal test. Conversely, the test showed 100% sensitivity and negative predictive values.* Salmonella* isolates were identified from 7 (7.07%) of 99 stool samples. Two-thirds of salmonellosis suspected patients received antibiotic treatment. However, only half of the confirmed salmonellosis patients were treated with appropriate antibiotics. All of the isolates were susceptible to ciprofloxacin and ceftriaxone but resistant to ampicillin.

**Conclusions:**

Majority of the patients who participated in this study were wrongly diagnosed using symptoms, clinical signs, and tube Widal test. Consequently, most of the patients received inappropriate treatment.

## 1. Introduction

Salmonellosis continues to be among the major causes of illnesses and deaths around the globe particularly in developing countries [1–3].* Salmonella enterica* subspecies I bacteria are the causes of the disease and over 93 million new infections worldwide each year [4]. World Health Organization (WHO) estimated 3 million* Salmonella*-associated deaths globally per year [5]. Diseases caused by* Salmonella* in humans are gastroenteritis or nontyphoidal salmonellosis (by* Salmonella Typhimurium* or* Salmonella Enteritidis*) [4] and typhoid fever (by* Salmonella* Typhi or Paratyphi) [6]. Global nontyphoidal salmonellosis burden estimate indicated 155,000 deaths each year [4]. In Sub-Saharan Africa, the invasive nontyphoidal salmonellosis often causes life-threatening bacteremia for about 1.9 million immunosuppressed individuals [7, 8].

Despite the high global burden of salmonellosis, well-established diagnosis and treatment have only recently begun to advance [7]. Since the clinical pictures of the disease are nonspecific; confirmed diagnosis relies on the gold standard test (culture) [9]. But culture is expensive, labor-intensive, and time-consuming [10]. In many typhoid fever endemic areas where culture facilities are not available, Widal test is considered to be the only quick diagnostic tool [11]. Though the Widal test is no longer being used as a diagnostic tool in developed nations due to its low sensitivity and specificity, it is still commonly in use in developing countries such as Ethiopia [9, 11]. Widal test is the rapid and affordable diagnostic assay available in Africa and it has been extensively employed since typhoid fever is endemic in most of the continent [12]. In the case of nontyphoidal salmonellosis, clinical signs and symptoms are still the main means of diagnosis [13].

Reports have shown increased typhoidal salmonellosis cases resistant to many drugs such as fluoroquinolones. In countries with a higher incidence of multidrug-resistant typhoidal* Salmonella* isolates, susceptibility to ciprofloxacin and other fluoroquinolones is reported between 44% and 59% [14]. Based on the National Antimicrobial Resistance Monitoring System survey from 2005 to 2006, 84% of nontyphoid* Salmonella* clinical isolates displayed multidrug resistant phenotype and 4.1% of the isolates showed reduced susceptibility to cephalosporins in the United States of America [15]. A surveillance study in Europe from 2000 to 2004 on nontyphoid* Salmonella* also showed that 15% of the isolates displayed multidrug resistant phenotype and 20% of the isolates showed resistance to nalidixic acid [16].

In Ethiopia, the diagnosis of typhoidal and nontyphoidal salmonellosis has been carried out using the Widal test and clinical pictures, respectively [17]. But the importance of both diagnostic methods has been strongly criticized [11]. Hence, evaluation of the effectiveness of these methods in the diagnosis of salmonellosis against the gold standard method is necessary for better patient outcomes. The main objective of this study was to examine the level of agreement between results of Widal test and clinical examinations with the results of culture method in the diagnosis of salmonellosis. In addition, the study aimed at assessing the appropriateness of treatment for the diagnosis and antimicrobial susceptibility pattern of* Salmonella* isolates.

## 2. Materials and Methods

### 2.1. Study Area, Sample Size, and Sampling Procedure

A cross-sectional study was carried out on salmonellosis suspected patients who visited Selam (public) and Alem Tena (private) clinics in Addis Ababa, Ethiopia, from August 2015 to March 2016. The sample size was determined using a single population proportion formula, *n* = *p*(1 − *p*)(*Zα*/2)2/*d*2 [18] with the following assumptions: *Zα*/2 = 1.96 for the standard scale of 95% level of confidence, level of precision (*d*) = 5%, and *p* = 0.5 since there was no previous study carried out on this issue in the current study health facilities. Ten percent nonresponse rate was considered. Study subjects who visited the two clinics and fulfilled the eligibility criteria were enrolled after receiving their written consent. Though the calculated sample population was 423, only 387 eligible and willing patients participated in the current study. Case history study was conducted on 367 patients since the remaining 20 patients' record could not be found in the study clinics. Sociodemographic data was gathered using structured and pretested questionnaire and patients' clinical records. All the febrile (*n* = 288) and diarrheic (*n* = 99) patients provided blood and stool samples, respectively. Stool specimens were collected using labeled wide-mouthed plastic containers and clean wooden applicator sticks. Venous blood samples, 8–10 ml from adults and 3–5 ml from children, were collected aseptically. All of the samples were collected during the first day of patients' examination in the study clinics.

### 2.2. Sample Analysis

#### 2.2.1. Blood Samples Analysis Using Widal Test

The Widal tube agglutination test (titration) was performed as described previously [9] using serial dilution of serum samples in 0.95% fresh saline preparation from 1 : 20 to 1 : 640 for anti-TO and anti-TH separately. Subsequently, a drop of O and H antigens was added to all test tubes. Antibody titer > 1 : 80 for anti-TO and >1 : 160 for anti-TH were taken as a cut-off value to indicate a recent infection with typhoid fever as per the manufacturer's manual (Chromatest Febrile Antigens kits, Linear Chemicals, Spain).

#### 2.2.2. Bacteriological Investigation of Blood Sample

All blood samples (*n* = 288) were collected before administration of any drug and after checking that patients did not take any form of antibiotics at least for the last 2 weeks. The bacteriological examination was carried out based on standard procedures [19, 20] with minor modifications. Briefly, blood samples were immediately inoculated into tryptic soy broth (Abtek USP, Abtek Biologicals Ltd., England) and transported to the Ethiopian Public Health Institute (EPHI) laboratory within 2–4 hours of collection. Upon arrival, culture bottles were incubated aerobically at 37°C for 5–7 days. Examination of incubated broths for growth indicators was conducted on daily basis and those showing growth were subcultured onto MacConkey (Oxoid, England) and blood (Oxoid, England) agar plates. The plates were incubated at 37°C in 5% CO_2_ incubator for a minimum of 48 hours and examined daily for the presence of distinct colonies. Bacterial identification was carried out based on colony morphology and Gram staining characteristics. Identified* Salmonella* isolates were further confirmed using biochemical, carbohydrates fermentation, and serotyping analysis.

#### 2.2.3. Stool Sample Analysis

Stool samples (*n* = 99) were submitted to the EPHI laboratory in sterile and leak-proof containers within 2 hours of collection and processed immediately according to the established protocol [21]. Approximately 1.5–2 grams of stool, preferably with blood and mucus, was mixed with Selenite F (TM Media, India) enrichment broth media and incubated for 24 hours at 37°C. Subculturing was carried out on MacConkey and* Salmonella*-Shigella (SS) agars (Oxoid, England) at 37°C for 24 hours. Non-lactose fermenter colonies on MacConkey and SS agars with black center on SS agar were subjected to biochemical, carbohydrates fermentation, and serotyping tests.

#### 2.2.4. Biochemical, Carbohydrate Fermentation, and Serotyping Tests

Presumptive* Salmonella* colonies were subjected to biochemical and carbohydrates fermentation tests including triple sugar iron agar (TSIA) (TM Media, India), lysine iron agar (LIA) (Oxoid, England), indole (TM Media, India), citrate (TM Media, India), and urease (Oxoid, England) tests as described earlier [19, 20, 22]. Poly antisera* Salmonella* O, antiserum poly A-I, and Vi (Difco™* Salmonella* O, BD, Ireland) were used during serotyping for confirmation. Colonies showed an alkaline slant with acid butt and hydrogen sulfide production on TSIA, positive for lysine on LIA, positive for citrate utilization on Simmons' citrate agar, negative for urea hydrolysis on urea agar, and negative for indole test on SIM medium and that agglutinate on tube agglutination test with* Salmonella* polyvalent antisera was considered as* Salmonella* isolate.

#### 2.2.5. Antimicrobial Susceptibility Test

All isolates were subjected to antimicrobial susceptibility test using Kirby-Bauer disk diffusion method. The bacterial suspension was prepared by taking 4-5 pure isolated colonies into trypticase soy yeast broth (Abtek Biologicals Ltd., England) and adjusted to 0.5 McFarland turbidity standards [23]. The suspension was transferred to Mueller-Hinton agar plate using a sterile cotton swab. Plates were uniformly inoculated by rubbing the swab against the entire agar surface and rotating plates at about 60 degrees 3 times. Antibiotic-impregnated disks were applied to the surface of the inoculated plates using sterile forceps and incubated aerobically at 37°C for 24 hours. Antibiotics were selected based on common prescription trend and availability of antibiotic disks.* Salmonella* isolates were tested for antibiotics (Oxoid, England) including ampicillin (AMP, 10 *μ*g), ceftriaxone (CRO, 30 *μ*g), chloramphenicol (C, 30 *μ*g), and ciprofloxacin (CIP, 5 *μ*g).

#### 2.2.6. Quality Control and Data Analysis

Sterility and performance tests were performed for all media prior to the tests.* S.* Typhimurium (ATCC 13311),* Escherichia coli* (ATCC 25922), and* Staphylococcus aureus* (ATCC 25923) were used as quality control species. Data were double entered, cleaned, and analyzed using Microsoft Excel Spreadsheet version 2016 16.0.6741.2048.

#### 2.2.7. Ethical Considerations

Ethical clearance was obtained from Institutional Board of Review of St. Paul's Hospital Millennium Medical College (protocol no. P. M23/72, on 17 February 2015). Written informed consent was obtained from all study participants. Participants also gave their written agreement that the research result will be published with keeping their identity anonymous. Salmonellosis suspected patients with antimicrobial treatment history within two weeks were excluded from the study. Information obtained during the course of the study was kept confidential and communicated to the clinics.

## 3. Results

### 3.1. Sociodemographic Characteristics of the Study Participants

Clinical records were found for 367 of salmonellosis suspected patients. The remaining 20 patients were registered as referral cases from other health facilities for Widal test purpose only. Among patients with complete records, 5.44% (*n* = 20) and 2.72% (*n* = 10) were under 10 and over 70 years, respectively. About 37% (*n* = 135) of the patients were in 21–30 years of age group. Majority of the patients were from economically productive age group (18–49 years). Close to 60% (*n* = 220) of the participants were males.

### 3.2. Diagnosis of Salmonellosis

According to the reviewed clinical records of the study clinics, the diagnosis of salmonellosis is carried out mainly based on clinical signs and symptoms. Of the total diseases listed as primary and secondary diseases, acute febrile illness was the most frequently mentioned disease (*n* = 208, 39.62%) as shown in Table 1. Nontyphoidal salmonellosis was the second common disease (*n* = 72, 13.71%) of all diseases documented. Typhoid fever is reported as the second (*n* = 43, 11.72%) and the least (*n* = 4, 1.09%) common disease under the primary and secondary diseases categories, respectively. In general, typhoid fever is among the top three common diseases recorded in the current study. Infectious diseases showing similar clinical signs and symptoms with typhoid fever such as acute febrile illness and unspecified infectious diseases were also the frequently listed diseases.

### 3.3. Widal Test, Culture, and Serotyping

Of 288 blood samples examined using tube Widal test, 51.39% (*n* = 148) were positive for O, H, or both antigens as tabulated in Table 2. Antibody titers of ≥1 : 80 for anti-TO and ≥1 : 160 for anti TH were taken as a cut-off value to indicate recent infection of typhoid fever. In the current study, tube Widal test demonstrated 100% sensitivity as well as negative predictive values for both O and H antigens. Conversely, it indicated very poor specificity (0.68%) and positive predictive values (48.78%). There was a good level of agreement (Kappa = 0.62) in detecting both antigens. Out of 148 blood samples positive for O, H, or both antigens, only 1 sample yielded* Salmonella* isolate. No* Salmonella* isolate was identified from Widal test negative blood samples. Among the 99 stool sample cultures, 7 yielded* Salmonella* isolates. Of 387 cultured blood and stool samples, only 8 produced* Salmonella* isolates. Six of the isolates were identified from diarrheic/dysentery patients and the remaining 2 were from acute febrile illness and unspecified infectious disease patients. All* Salmonella* isolates were confirmed using serotyping.

### 3.4. Antibiotic Treatment and Susceptibility Patterns

Nearly 67% (*n* = 245) of salmonellosis suspected patients were treated with one or more antibiotics. Among patients who received antibiotics treatment, 49.8 (*n* = 122), 25.3 (*n* = 62), 9.8 (*n* = 24), and 7.76% (*n* = 19) took ciprofloxacin, doxycycline, metronidazole, and cotrimoxazole, respectively, as shown in Table 3. All the isolates were susceptible to ciprofloxacin and ceftriaxone but resistant to ampicillin.

## 4. Discussion

Majority of the participants of the current study were from economically productive age group (18–49 years). This finding is in disagreement with studies that reported that less than 5 years of age is the highly affected segment of the population [24, 25]. Putting the diagnosis in a broad category like acute febrile illness, diarrhea/dysentery, and unspecified infectious diseases rather than naming specific disease is an indication of lack of clear picture of the disease among physicians.

Culture is the gold standard diagnostic method for* Salmonella* infection. But it is often unavailable or less commonly applied in low- and middle-income countries like Ethiopia due to time and cost constraints [1, 26]. Instead, Widal test is still widely used for typhoid fever suspected patients diagnosis despite the fact that it has poor predictive outcome [27]. Clinical signs and symptoms are still the routine diagnostic methods for nontyphoidal salmonellosis in developing countries [28]. Nonspecific names of diseases such as acute febrile illness, diarrhea/dysentery, and unspecified infectious diseases are frequently listed by physicians which might be due to the lack of confirmatory diagnostic tools.

Almost half of the blood samples were positive either for O, H, or both* Salmonella* antigens during the Widal test. This result is similar to a report from India [29], lower than from Ethiopia [30], but higher than from Kenya [31]. Culture positive results of samples collected from typhoidal salmonellosis suspected patients in the current and other studies [29–31] are very low compared with Widal test results. This might suggest that there has been overdiagnosis of typhoid fever due to the very poor specificity of Widal test [9, 11, 27, 29].

In this study, the identification of* Salmonella* from both blood and stool samples is lower than reports from Ethiopia [3, 17, 30]. The variation might be partly attributed to difference in study settings since the current study is conducted at clinics but the previous studies were carried out at hospitals where prolonged febrile illness patients are found. The very poor positive predictive value and low specificity of Widal test results of the current research are in agreement with previous studies in Ethiopia [17, 30] and other countries [29, 32]. Since positive predictive value is considered as one of the most important clinical diagnostic tools [17, 30], very low positive predictive value in the current research indicates that Widal test was not reliable for the diagnosis of the disease in the study settings. The very high false positive result of the Widal test in this research is an indication that patients are wrongly diagnosed and treated as typhoid fever patients. This inappropriate treatment based on tube Widal test results and clinical signs and symptoms might have created unnecessary treatment costs, pressure on the normal flora of patients, and favorable condition for antimicrobial resistance strains development [4, 30]. Most importantly, highly fatal diseases of febrile nature might be missed out and ultimately lead to poor prognosis.

The current prevalence of nontyphoidal salmonellosis as confirmed by culture and serotyping is comparable with previous studies in Ethiopia [33] and in Sub-Saharan Africa [34]. The relatively higher prevalence of nontyphoidal salmonellosis compared with typhoidal salmonellosis in this study is in agreement with the report that suggested the proportion of typhoidal* Salmonella* infection tends to decline since 1970s while nontyphoidal* Salmonella* infection shows a relative increase in Ethiopia [33]. The current nontyphoidal* Salmonella* infection prevalence might be lower than its actual occurrence since many patients in developing countries do not seek medical attention for less severe gastroenteritis. They are unable to cover medical costs even for severe cases. Widespread malnutrition, higher HIV-AIDS prevalence, close relationship with animals, the common practice of backyard animal slaughtering, unhygienic food and water handling, raw meat consumption habits, and population crowd are suggestive of its higher prevalence than reported in the current study.

Nowadays, drug resistant* Salmonella* infection is one of the critical problems of the globe [35]. As demonstrated by research results [2, 36–38] and summarized by the recent review [39], there is an increasing prevalence of drug resistant* Salmonella* isolates in Ethiopia. The resistance of all* Salmonella* isolates against ampicillin in the current study, which is higher than reports in Ethiopia [2, 36, 38, 39], is an indication of worsening situation of antimicrobial resistance* Salmonella* in the country.

Due to the increase in the resistance against ampicillin, ciprofloxacin continues to be widely used globally in the treatment of salmonellosis [1]. Matching with the global trend, this study revealed that ciprofloxacin is the most frequently prescribed drug followed by doxycycline and metronidazole in the study clinics. This treatment regimen seems appropriate since all* Salmonella* isolates were susceptible to ciprofloxacin. But only half of the confirmed salmonellosis patients were treated with appropriate antibiotics. Vigilant use of ciprofloxacin is needed since resistant isolates are reported from countries in Europe, Asia, Africa, and the United States [8].

## 5. Conclusions

The general prevalence of salmonellosis reported in the current study is low. This low culture proven salmonellosis prevalence demonstrated that the vast majority of patients were wrongly diagnosed using clinical signs and symptoms and Widal test. The wrong diagnosis was also followed by incorrect treatment that might have had a grave consequence on the patients' prognosis. This situation might have also posed a high risk for antimicrobial resistance strains development. Since the Widal test is found to be inappropriate as a diagnostic tool for typhoidal salmonellosis in endemic areas like Ethiopia, it should be either replaced with more accurate tests or followed by alternative diagnostic tools. The resistance of all* Salmonella* isolates against ampicillin is an indication that ampicillin is no more effective in the treatment of salmonellosis. Vigilant prescription of ciprofloxacin for salmonellosis patients is encouraged. The nationwide in-depth investigation regarding the prevalence of the disease, circulating serotypes, their antimicrobial susceptibility patterns, and appropriate diagnostic options is strongly recommended.

## Figures and Tables

**Table 1:** List of diseases and treatment trend (with antibiotics and/or anthelmintics) in selected clinics, Addis Ababa, Ethiopia.

Name of the diseases	Category	As primary disease		As secondary disease		Total	
		Freq.	%	Freq.	%	Freq.	%
Acute febrile illness	Total	185	50.40	23	6.27	208	39.62
	Treated	129	69.73	19	82.61	148	71.15

Diarrhea/Dysentery	Total	53	14.44	19	5.18	72	13.71
	Treated	45	84.91	3	15.79	48	66.67

Typhoid fever	Total	4	1.09	43	11.72	47	8.95
	Treated	4	100	40	93.02	44	93.62

Unspecified infectious diseases	Total	39	10.63	6	1.63	45	8.57
	Treated	29	74.36	1	16.67	30	66.67

Dyspepsia	Total	17	4.63	12	3.26	29	5.52
	Treated	15	88.24	9	75.00	24	82.76

Intestinal parasitism	Total	5	1.36	22	5.99	27	5.14
	Treated	5	100	19	86.36	24	88.89

Urinary tract infection	Total	10	2.72	15	4.09	25	4.76
	Treated	10	100	14	93.33	24	96.00

Respiratory tract diseases	Total	12	3.27	10	2.72	22	4.19
	Treated	11	91.67	9	90.00	20	90.91

Others	Total	23	6.27	27	7.37	50	9.52
	Treated	18	78.26	25	92.59	43	86

No diagnosis recorded	Total	19	5.18	190	51.77	18	100
	Treated	2	10.53	32	16.84	1	5.56

Total	Patients Recorded	367	100	367	100	367	100
	Diagnosis recorded	348	94.82	177	48.23	525	100
	Treated	268	73.02	171	46.59	439	83.62

**Table 2:** Widal test result (*n* = 288) in selected clinics, Addis Ababa, Ethiopia.

Test	Result	Freq.	%
Widal test against *Salmonella* H antigen	−Ve	165	57.29
	+Ve	123	42.71

Widal test against* Salmonella* O antigen	−Ve	168	58.33
	+Ve	120	41.67

Widal test against both H and O antigens of* Salmonella*	Both −Ve	140	48.61
	Both +Ve	95	32.99
	+ve for either O or H	148	51.39
	H +Ve but O −Ve	25	8.68
	O +Ve but H −Ve	28	9.72

**Table 3:** Antimicrobials prescribed for salmonellosis suspected patients in selected clinics, Addis Ababa, Ethiopia.

Treatment	Frequency	Percentage
Patients who received antimicrobial treatment	245	66.76
Ciprofloxacin	122	49.8^*∗*^
Doxycycline	62	25.3^*∗*^
Metronidazole	24	9.8^*∗*^
Cotrimoxazole	19	7.76^*∗*^
Amoxicillin	5	2.04^*∗*^
Ceftriaxone	4	1.63^*∗*^
Other antibiotics	9	3.67^*∗*^
Patients who received no antimicrobial treatment	122	33.24
Total	367	100

^*∗*^Percentage of patients who received antimicrobial treatment.
